# Novel Photosensitizers Trigger Rapid Death of Malignant Human Cells and Rodent Tumor Transplants via Lipid Photodamage and Membrane Permeabilization

**DOI:** 10.1371/journal.pone.0012717

**Published:** 2010-09-15

**Authors:** Mikhail M. Moisenovich, Valentina A. Ol'shevskaya, Tatyana I. Rokitskaya, Alla A. Ramonova, Roza G. Nikitina, Arina N. Savchenko, Victor V. Tatarskiy, Mikhail A. Kaplan, Valery N. Kalinin, Elena A. Kotova, Oleg V. Uvarov, Igor I. Agapov, Yuri N. Antonenko, Alexander A. Shtil

**Affiliations:** 1 Biological Department, Moscow State University, Moscow, Russia; 2 Nesmeyanov Institute of Organoelement Compounds, Moscow, Russia; 3 Belozersky Institute of Physico-Chemical Biology, Moscow State University, Moscow, Russia; 4 Medical Radiological Research Center, Obninsk, Russia; 5 Blokhin Cancer Center, Moscow, Russia; 6 Prokhorov Institute of General Physics, Moscow, Russia; 7 Shumakov Research Center of Transplantology and Artificial Organs, Moscow, Russia; Technical University Munich, Germany

## Abstract

**Background:**

Apoptotic cascades may frequently be impaired in tumor cells; therefore, the approaches to circumvent these obstacles emerge as important therapeutic modalities.

**Methodology/Principal Findings:**

Our novel derivatives of chlorin e_6_, that is, its amide (compound **2**) and boronated amide (compound **5**) evoked no dark toxicity and demonstrated a significantly higher photosensitizing efficacy than chlorin e_6_ against transplanted aggressive tumors such as B16 melanoma and M-1 sarcoma. Compound **5** showed superior therapeutic potency. Illumination with red light of mammalian tumor cells loaded with 0.1 µM of **5** caused rapid (within the initial minutes) necrosis as determined by propidium iodide staining. The laser confocal microscopy-assisted analysis of cell death revealed the following order of events: prior to illumination, **5** accumulated in Golgi cysternae, endoplasmic reticulum and in some (but not all) lysosomes. In response to light, the reactive oxygen species burst was concomitant with the drop of mitochondrial transmembrane electric potential, the dramatic changes of mitochondrial shape and the loss of integrity of mitochondria and lysosomes. Within 3–4 min post illumination, the plasma membrane became permeable for propidium iodide. Compounds **2** and **5** were one order of magnitude more potent than chlorin e_6_ in photodamage of artificial liposomes monitored in a dye release assay. The latter effect depended on the content of non-saturated lipids; in liposomes consisting of saturated lipids no photodamage was detectable. The increased therapeutic efficacy of **5** compared with **2** was attributed to a striking difference in the ability of these photosensitizers to permeate through hydrophobic membrane interior as evidenced by measurements of voltage jump-induced relaxation of transmembrane current on planar lipid bilayers.

**Conclusions/Significance:**

The multimembrane photodestruction and cell necrosis induced by photoactivation of **2** and **5** are directly associated with membrane permeabilization caused by lipid photodamage.

## Introduction

Photodynamic therapy (PDT), a modality that evokes cell damage after illumination in the presence of a photosensitizing agent, is gaining momentum in treatment of a variety of human diseases including cancer [1]–[3]. Growing number of clinical situations in which PDT has demonstrated its efficacy makes the design of new generations of photosensitizers all the more necessary. Tetrapyrrole containing compounds whose macrocyclic moiety plays a key role in generating cytotoxic reactive oxygen species (ROS) upon light illumination (LI), are especially suitable for chemical modifications of the coordination sphere and at the periphery of the macrocycle. In particular, introduction of metal cations such as Pd and Sn into the coordination sphere of tetrapyrrolic compounds conferred an increased photosensitizing potency [4]–[6]. Phenol [7], [8] and lipophilic groups [9] as the peripheral substituents in chlorins have been reported to be therapeutically relevant. Furthermore, conjugation of boron containing polyhedra (carboranes), a modification aimed primarily at compounds for boron neutron capture therapy, yielded agents more efficient in PDT than their boron-free counterparts. Indeed, the water soluble 1,3,5,8-tetramethyl-2,4-divinyl-6(7)–[2-(1-carba-*closo-*dodecaboran-1-yl)methoxycarbonylethyl]-7(6)-(2-carboxyethyl)porphyrin Fe(III) containing 1-carba-*closo*-dodecaborate anion demonstrated high potency in sensitizing rat M-1 sarcoma to PDT whereas at equimolar concentrations the initial boron-free protohemin IX was inert [10]. Chlorins are advantageous as natural photosensitizers due to light absorption in the long wavelength spectral region [11], [12]; therefore, tumor photodamage is expected to be deeper whereas the overlying tissues are spared. We synthesized novel boronated conjugates of chlorin e_6_ with 1-carba-*closo-*dodecaborate anion [13]. Vicente and colleagues [14] prepared a boronated chlorin based on the synthetic 5,10,15,20-tetrakis(pentafluorophenyl)chlorin derivative and 1-mercapto-*o*-carborane. These compounds demonstrated low-to-null dark toxicity, good intratumoral accumulation and an ability to kill cultured cells and tumor xenografts after photoactivation. Thus, varying the characteristics of the macrocycle and the boron substituents, one may obtain modified photosensitizers with desirable lipophilic, hydrophilic and amphiphilic properties, boron content, intracellular distribution, etc. [15], [16]. These characteristics are key factors that determine the mechanisms of tumor cell death upon photoactivation [17].

In the present study we analyze the mechanism of antitumor effect of two derivatives of chlorin e_6_, namely, chlorin e_6_ 13(1)-N-(2-aminoethyl}amide-15(2), 17(3)-dimethyl ester (chlorin e_6_ amide; compound **2**) and its novel boronated congener chlorin e_6_ 13(1)-N-{2-[N-(1-carba-*closo*-dodecaboran-1-yl)methyl]aminoethyl}amide-15(2), 17(3)-dimethyl ester sodium (compound **5**). PDT of transplanted syngeneic tumors demonstrated a substantially higher efficacy of **5** compared with chlorin e_6_ and **2**. The boronated compound **5** was capable of penetrating through planar lipid membranes in the ionic form whereas the boron-free **2** was not. Laser confocal microscopy showed that **2** and **5** accumulated in Golgi cysternae, endoplasmic reticulum (ER) and some lysosomes. Illumination of cells with red light rapidly triggered intracellular ROS generation followed by necrosis. In a cell free system, photoactivation of **2** or **5** caused damage to membranes of artificial vesicles manifested in their leakage, strongly suggesting that lipid membrane permeabilization is a key factor of an instant cell death induced by the novel chlorin e_6_ based photosensitizers.

## Results

### Synthesis of photosensitizers

We used methylpheophorbide *a* (**1**; Figure S1) as starting compound for preparation of free base boronated chlorin e_6_ derivatives [18]. The synthesis proceeded via the formation of the amide derivative **2** obtained after the nucleophilic opening of the exocyclic ring in **1** with ethylenediamine. Alkylation of amino group in **2** with 1-trifluoromethanesulfonylmethyl-1-carba-*closo*-dodecaborate cesium **3** [19] in tetrahydrofuran in the presence of N,O-bis(trimethylsilyl)acetamide led to chlorin e_6_ 13(1)-N-{2-[N-(1-carba-*closo*-dodecaboran-1-yl)methyl]aminoethyl}amide-15(2), 17(3)-dimethyl ester cesium **4**. For biological studies the sodium salt, chlorin e_6_ 13(1)-N-{2-[N-(1-carba-*closo*-dodecaboran-1-yl)methyl]aminoethyl}amide-15(2), 17(3)-dimethyl ester sodium **5** was obtained from cesium salt **4** (Figure S1). The procedures of synthesis and the properties of compounds are presented in File S1 (Supporting Information).

We measured quantum yield of singlet oxygen production by compounds **2**, **5** and chlorin e6 using dimethylanthracene photooxidation (DMA) in dimethyl sulfoxide (DMSO) solution. Figure S2 shows the time course of DMA fluorescence at 426 nm upon continuous light exposure (680 nm laser diode) in the presence of 1 µM of each compound. The quantum yields of singlet oxygen generation were estimated using the slopes of the plots [20], [21]. The quantum yields of compounds were very close: 68%, 61%, and 76% for **5**, **2** and chlorin e_6_, respectively, as compared with the reference compound tetrasulfonated zinc phthalocyanine (ZnPcS_4_) determined under the same conditions. The quantum yield of ZnPcS_4_ in DMSO was shown to be 68% [22].

### Antitumor efficacy of photosensitizers

We tested the dark cytotoxicity of chlorin e_6_ and compounds **2** and **5** using McA 7777 rat liver epithelium, Rat-1 and REF fibroblasts, C6 rat glioma, MCF-10A human breast epithelium, HCT116 human colon carcinoma and human donor lymphocytes. Cells were continuously exposed to various concentrations (up to 50 µM) of chlorin e_6_, **2** or **5** for 72 h. Within this range of concentrations the compounds were readily soluble in water. No growth retardation or cell death was registered in any of tested cell lines in the course of treatment in the dark.

To study the photosensitizing potency of chlorin e_6_, **2** and **5** in cellular PDT, we loaded C6 glioma cells with 0.1 µM of each compound for 15 min followed by drug withdrawal and LI of cells (see Materials and Methods for details). The phototoxic effect was clearly detectable immediately after the completion of LI. The cells lost their polygonal shape and became rounded. Flow cytometry assisted analysis revealed that in cultures loaded with 0.1 µM of **5**, 76±8% of cells (mean ± S.D., results of 5 experiments) were propidium iodide (PI)-positive as soon as 5 min after LI. More than 98% of cells were PI-positive by 20 min post LI whereas this parameter was <5% in cells exposed to **5** alone or after LI in the absence of **5**. In cells loaded with higher concentrations of the photosensitizer (up to 1 µM) >99% of cells showed PI staining immediately after the completion of LI. Similar results were obtained with **2** whereas chlorin e_6_ was without effect at the tested concentrations (not shown). Also, at equimolar concentrations **2** and **5** were more efficacious than chlorin e_6_ in PDT of HCT116 and MCF-7 cells (not shown). Thus, the high photodamaging potency of **2** and **5** at the concentrations that caused no dark toxicity suggested that these compounds might be good candidates for testing in animal models.

**Figure 1:**
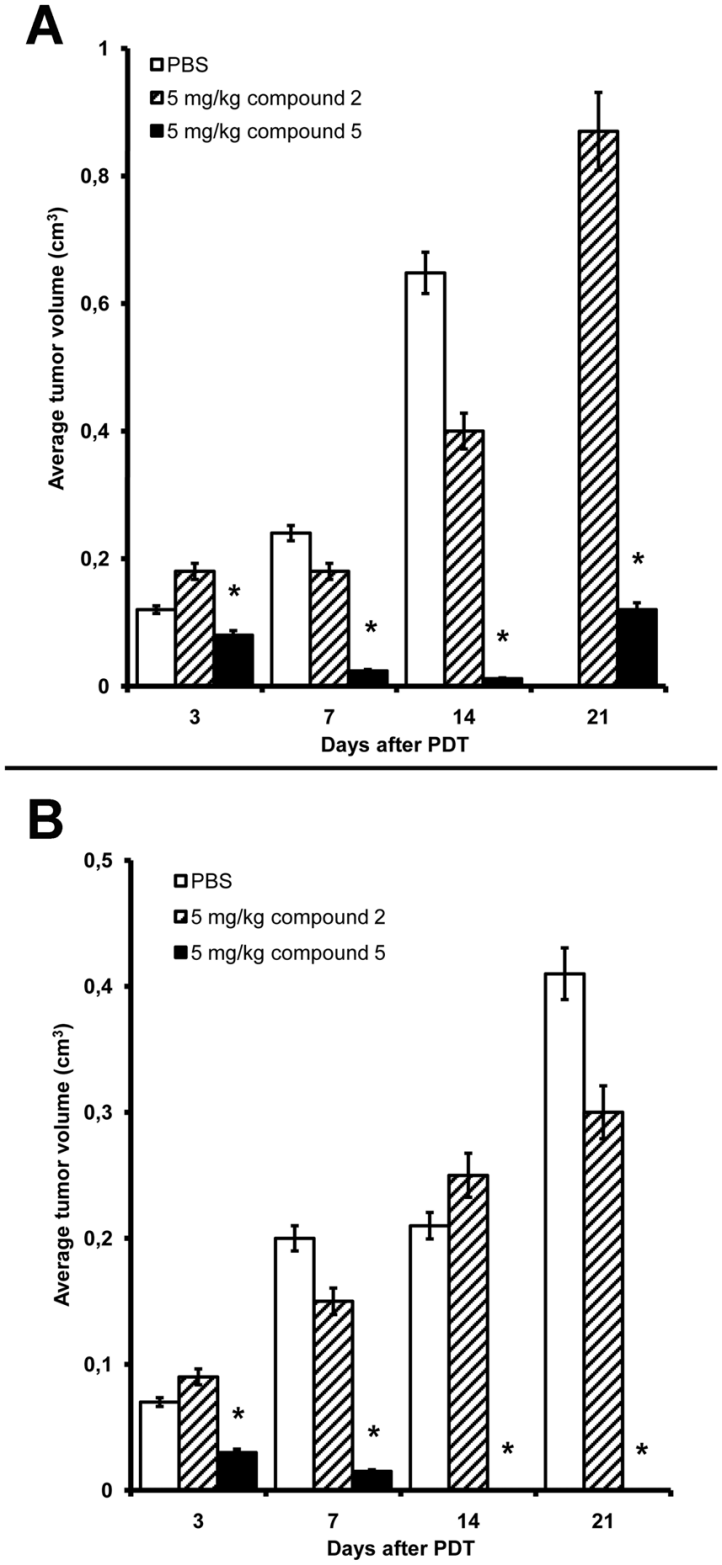
Potency of compounds 2 and 5 as antitumor photosensitizers. Mice bearing s.c. transplant of B16 melanoma (A) or rats bearing s.c. transplant of M-1 sarcoma (B) were injected i.p. with PBS or 5 mg/kg of **2** or **5**. Ninety minutes after injection the tumors were illuminated with red light (see MATERIALS AND METHODS). The tumor volume was measured at indicated time intervals. One representative experiment out of a total of three is shown. *p<0.01 between the ‘compound **5**’and the ‘compound **2**’ groups.

We investigated the antitumor efficacy of chlorin e_6_, **2** and **5** in PDT of transplanted murine B16 melanoma [23] and rat M-1 sarcoma [24]. After the tumor nodules reached ∼3–5 mm in diameter, animals were divided into 6 cohorts per each species (10 animals per group). Mice in the group 1 were injected with phosphate buffered saline pH 7.2 (PBS), mice in the group 2 were given LI in the absence of photosensitizers. Mice in groups 3–4 were injected with 5 mg/kg of either **2** or **5**, respectively; no LI was administered in these groups. Mice in groups 5–6 were injected with 5 mg/kg of **2** or **5**, respectively, followed by LI. Similar cohorts 1–6 were formed for rats bearing M-1 sarcoma transplants. In preliminary experiments we tested the range of tolerable concentrations of **5** in mice and rats. Compound **5** evoked no acute toxicity (as judged by unaltered behavior, nutritional habits and hair cover of animals, and by unchanged blood cell count) at the doses as high as 300 mg/kg injected i.p. At these concentrations **5** was soluble in water. Therefore, we tested several doses of **5** below 300 mg/kg for PDT; 5 mg/kg was a dose at which the therapeutic effect was highly reproducible. As shown in Figure 1A, tumors in the group 1 (PBS) grew fast: by day 14 the mean increase of tumor volume was 10.8-fold. Similar rates of tumor growth were registered in groups 2–4 (not shown). By day 15 the animals in groups 1–4 were sacrificed. In striking contrast, tumor illumination of mice injected with **5** (group 6) dramatically decreased the size of melanoma nodules. By day 14 post PDT the tumors were barely palpable (Figure 1A). However, in the group 6 the melanoma nodules resumed growth by day 21 post PDT, although the rate of tumor re-growth was slower than in mock-treated animals. By day 30 post PDT mice in group 6 were sacrificed; no lung metastases were detectable on autopsy. The boron-free precursor of **5** (compound **2**) was significantly less efficient than **5**: PDT with 5 mg/kg of **2** (group 5) delayed tumor growth only partially (Figure 1A).

Compound **5** demonstrated also high efficacy in PDT of engrafted M-1 sarcoma. In all control cohorts of rats (groups 1–4) the tumors enlarged with time. In rats exposed to PDT with 5 mg/kg of **5** the tumors gradually shrank; no nodules were detectable 14 days post treatment (Figure 1B). All animals in this group were cured, with no signs of tumor recurrence for at least 60 days post PDT. In contrast, **2** at equal dose evoked little therapeutic effect. Overall, **5** demonstrated remarkably high photosensitizing potency in cell culture and in vivo. We therefore set out to investigate the mechanisms of cell death triggered by photoactivation of **5**.

### Multimembrane photodamage by compound 5

**Figure 2:**
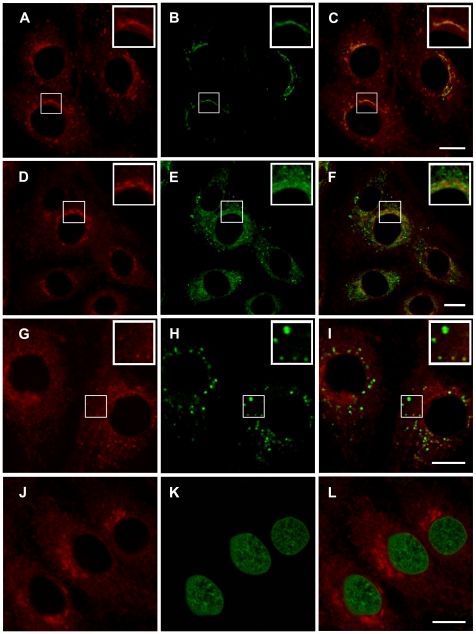
Intracellular distribution of compound 5. The C6 rat glioma cells were stained with the Golgi marker BODIPY FL C5–ceramide (A–C), the ER marker brefeldin A, BODIPY FL (D–F) the lysosomal dye LysoTracker*®*Green DND-99 (G–I), or the nuclei marker SYBR Green (J–L). Then 0.3 µM of **5** was added for 15 min. After removal of the drug cells were analyzed by confocal scanning microscopy. A, D, G and J: autofluorescence of **5**. B, Golgi marker; E, ER marker; H, lysosomal marker, K, nuclei marker, C, F, I and L: overlays of A and B, D and E, G and H and J and K, respectively. Shown are the optical sections at the midst of the nucleus. The experiments were performed at 37°C, 5%CO2. In A–I, insets in the upper right corners show Golgi, ER and lysosomes at a higher magnification. Bar, 10 µm.

Intracellular distribution of **5** was studied by laser confocal scanning microscopy. The drug entered the cells within the initial 30 sec. Fifteen min later **5** accumulated in the cytoplasm, predominantly in the perinuclear area (Figure 2). Cell labeling with organelle-specific fluorescent dyes demonstrated that substantial amounts of **5** were accumulated in Golgi cysternae (Figure 2A-C). Also, some amount of **5** was detected in ER. Furthermore, the tubular structures stained with **5** were associated with ER (Figure 2D-F). In addition, **5** was detectable in some (but not all) lysosomes (Figure 2G-I). Finally, we found diffuse cytoplasmic distribution of the photosensitizer. We found no significant amounts of **5** in the nuclei (Figure 2J-L). The signal of **5** in the nuclear area did not change during incubation in the dark for as long as 2 h (not shown).

**Figure 3:**
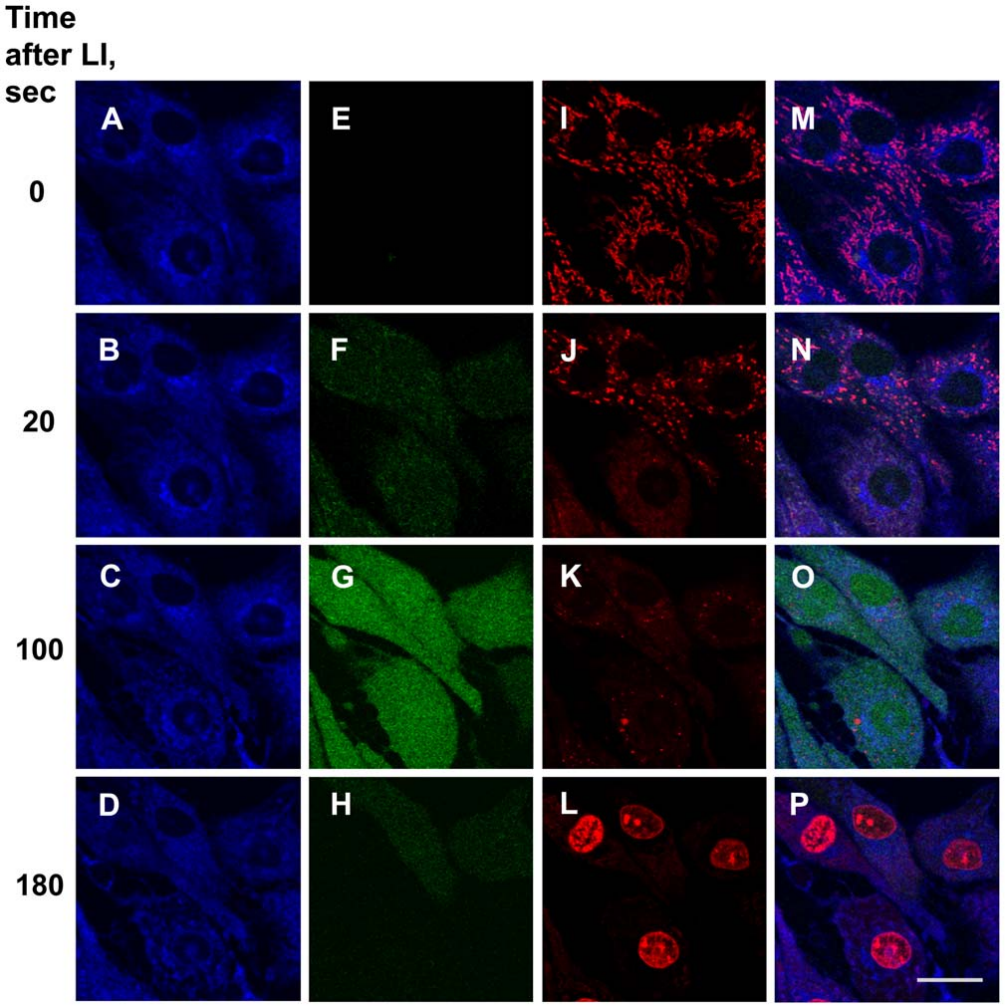
Time course of death-associated events after LI of cells loaded with compound 5. The C6 rat glioma cells were incubated with 1 µM of **5** for 15 min. at 37°C, 5%CO_2_. A–D: fluorescence of **5**. E–H: ROS generation detected with DCFH-DA (4 µM, 5 min.). I–K: changes of mitochondrial morphology and a decrease of Δ*Ψ_m_* detectable with MitoTracker Red CM-H_2_XRos (0.5 µM, 5 min.). L, staining with PI (15 µM). Fluorescence of MitoTracker Red CM-H_2_XRos and PI was detected in the same channel. M: overlay of A, E and I. N: overlay of B, F and J. O: overlay of C, G and K. P: overlay of D, H and L. Shown are optical sections. Bar, 20 µM.

Figure 3 shows the sequence of events that led to cell death after LI of cells loaded with **5**. As early as 20 sec post LI a weak but clearly detectable fluorescence of 2',7'-dichlorofluorescein (DCF), the oxidation product of the ROS indicator 2',7'-dichlorodihydrofluorescein diacetate (DCFH-DA) was observed (Figure 3; compare E and F). This phenomenon coincided with a drop of mitochondrial transmembrane electric potential Δ*Ψ_m_* (Figure 3; I vs J). The time dependent increase of DCF fluorescence was paralleled by the decrease of Δ*Ψ_m_* (Figure 3; G vs F and K vs J). Furthermore, a gradual decrease of fluorescence of LysoTracker was observed, suggesting that pH in lysosomes was neutralized (not shown). By 100 sec post LI Δ*Ψ_m_* dissipated. Finally, by 3 min post light exposure no fluorescence of the ROS indicator was observed (Figure 3H, P). The nuclei were stained with PI, indicating the loss of the plasma membrane integrity (Figure 3L, P). It is noteworthy that the nuclei retained their shape, and no signs of DNA degradation were visible. In striking contrast, in cells illuminated in the absence of **5** none of the above-mentioned phenomena, that is, intracellular ROS generation, dynamics of Δ*Ψ_m_* or PI staining, were detectable after 7 min post LI (not shown).

**Figure 4:**
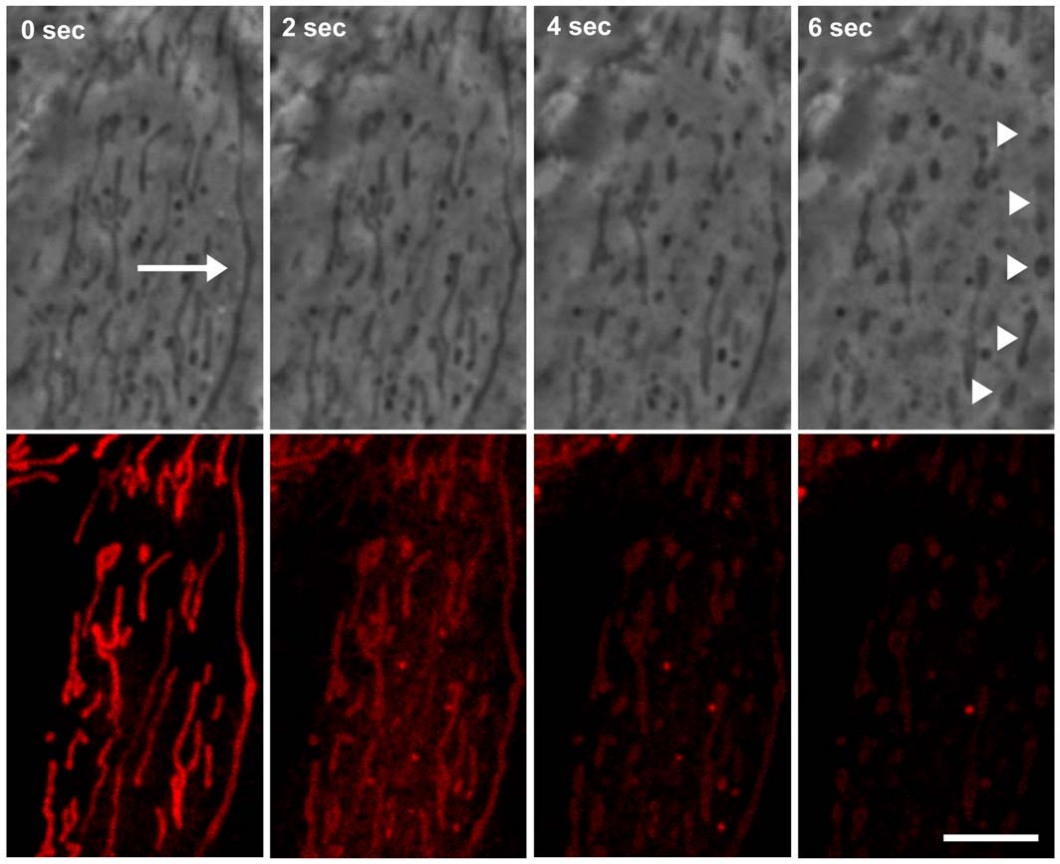
Fragmentation of mitochondria after PDT with compound 5. Top panel: phase contrast, bottom panel: fluorescence of MitoTracker Red CM-H_2_XRos. Time after LI is shown in upper left corners. Arrow, intact mitochondria; arrowheads, fragmented mitochondria. Bar, 5 µm.

Alterations of mitochondria occurred rapidly after LI in the presence of **5**. Fragmentation of mitochondria and a decrease of MitoTracker Red CM-H_2_XRos fluorescence were detectable within the initial seconds after LI (Figure 4).

Thus, monitoring of cell death by confocal microscopy revealed the following events: intracellular ROS generation concomitant with fragmentation of mitochondria, Δ*Ψ_m_* drop and subsequent loss of plasma membrane permeability for PI. Importantly, PDT caused photodamage of the organelles and the plasma membrane whereas nuclear fragmentation was absent. Essentially the same order of death-associated phenomena was detected after LI of HCT116 and MCF-7 cell lines loaded with **5** (not shown). These data indicated that necrosis was the primary mechanism of cell death upon photoactivation of **5**. The patterns of cell death upon photoactivation of **2** were similar to those observed for **5** (not shown).

**Figure 5:**
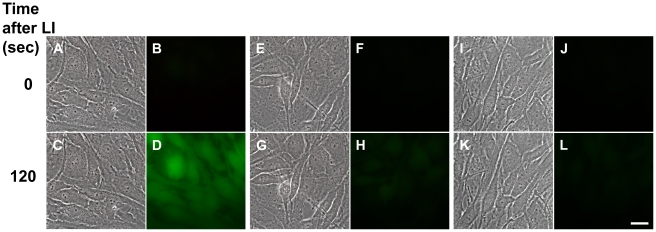
Sodium azide and trolox inhibit the increase of intracellular ROS level after PDT with compound 5. The C6 glioma cells were loaded for 15 min with DCFH-DA and **5** in the absence (A–D) or presence of 10 mM NaN_3_ (E–H) or 10 µM trolox (I–L). A, C, E, G, I and K, phase contrast; B, D, F, H, J and L, epifluorescence. Images were taken before (upper panel) or after (bottom panel) LI. Note green staining in D and its disappearance in H and L. Bar, 10 µm.

Next, we addressed the question of whether the DCF fluorescence observed after illumination in the presence of **5**, reflected intracellular ROS generation. LI of C6 glioma cells loaded with DCFH-DA and **5** caused bright green fluorescence (Figure 5). Importantly, both sodium azide and trolox blocked the increase of DCF fluorescence, reflecting ROS generation induced by LI in the presence of **5**. These results are in line with the observation that sodium azide, a known singlet oxygen quencher, suppresses the PDT-induced cellular ROS generation as measured by DCFH oxidation [25]. Trolox has been reported to quench singlet oxygen [26] and photosensitizer triplet states [27], [28] as well as to scavenge lipid peroxyl radicals [29].

### Lipid membrane permeabilization upon photoactivation of chlorin e_6_ derivatives

**Figure 6:**
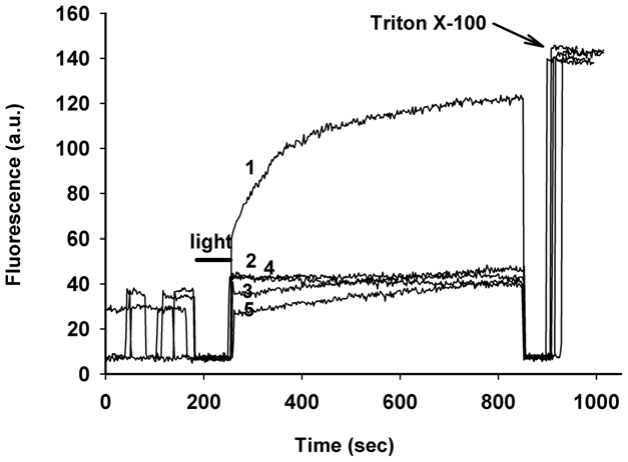
Oxidative photodamage of liposomes in the presence of compound 5. The SRB-containing EggPC liposomes were suspended in PBS and either mock-treated or exposed to red light in the presence of 350 nM of **5** alone or **5** and 10 mM NaN_3_. A line bar and an arrow indicate the time of LI and the addition of 0.1% Triton X-100, respectively. Liposome photodamage was registered as an increase of SRB fluorescence in the extra-liposomal milieu. Shown is one representative experiment out of 3 independent replicates.

To get insight into the mechanism of ROS-induced membrane photodamage we investigated the effects of chlorin e_6_ based photosensitizers on the integrity of artificial liposomes. Photodamage to liposomes loaded with the fluorescent dye sulforhodamine B (SRB) was monitored in a dye leakage assay. Inside intact liposomes SRB is self-quenched due to its high concentration (50 mm) whereas in permeabilized liposomes SRB redistributes into the buffer. Figure 6, curve 1 shows the time course of SRB fluorescence in the buffer after illumination of EggPC liposomes in the presence of 5. Mock-treated liposomes (intact, curve 2; liposomes exposed to 5 in the absence of light, curve 3; and liposomes illuminated in the absence of 5, curve 4) were used as a control. Illumination of liposomes in the presence of 350 nM of 5 rapidly caused leakage of SRB. The SRB fluorescence in the buffer reached the plateau within ∼200–300 sec. The intensity of fluorescence at the plateau was close to the complete dye efflux as determined by measurement of fluorescence after the addition of Triton X-100, the treatment that caused light-independent total liposome permeabilization. In striking contrast, in mock-treated liposomes SRB fluorescence did not exceed the basal level (Figure 6). These results indicated that the loss of liposome integrity required photoactivation of 5. Importantly, NaN_3_, a singlet oxygen quencher, abrogated liposome photodamage (Figure 6, curve 5). Similar results were obtained with 2 (not shown).

**Figure 7:**
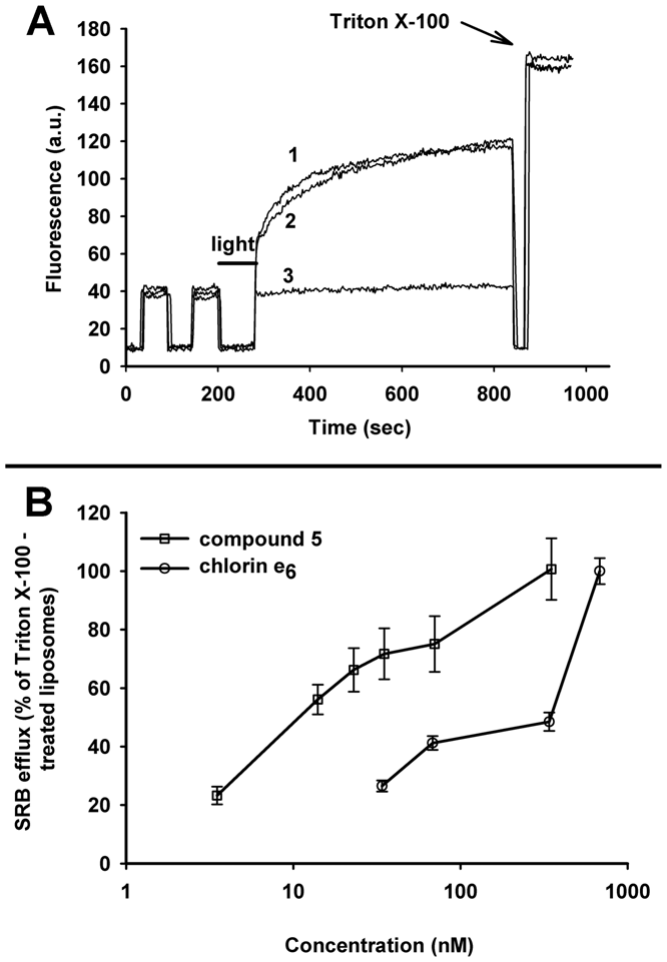
Time- and concentration dependence of liposome permeabilization. The SRB-loaded EggPC liposomes were suspended in PBS and illuminated in the presence of 35 nM of **5** (curve 1) or chlorin e_6_ (curve 2) (top panel; one representative experiment out of 4 with similar results). Bottom panel: dependence of SRB efflux (% at 100 s after LI) on indicated concentrations of each compound (bottom panel; mean ± S.D. of 3 experiments). Differences between the values of SRB efflux for the respective concentrations of compounds are statistically significant (p<0.01).

Furthermore, illumination of SRB-loaded liposomes for 1 min with 35 nM of **5** triggered a fast dye leakage whereas chlorin e_6_ at the same concentration caused no photodamage (Figure 7A). At 3 nM of **5** ∼20% of total dye content leaked at 100 s after LI, and more than one half of the dye was out of liposomes 100 sec after LI with 20 nM of **5**. In striking contrast, even with 600 nM of chlorin e_6_ the liposome photodamage was weaker than with 20 nM of **5**. At 600 nM of **5** all amount of SRB leaked from liposomes by 100 sec after LI (Figure 7B) whereas micromolar concentrations of chlorin e_6_ were required for total liposome permeabilization (not shown). Compound **2** showed similar potency as **5** in liposomal photodamage (not shown).

**Figure 8:**
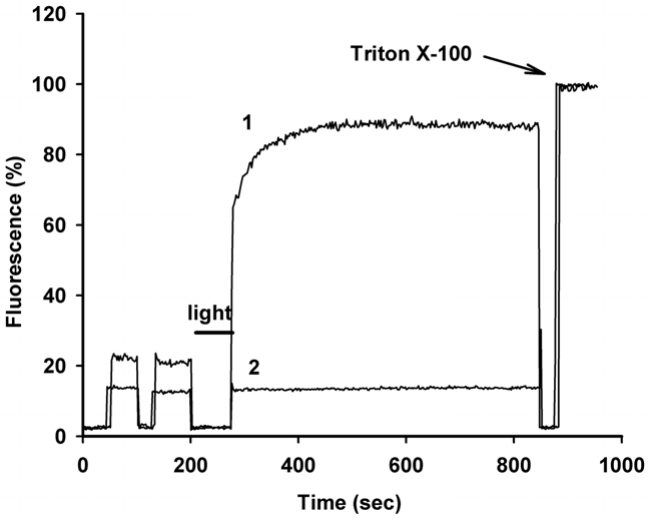
Dependence of liposome photodamage on lipid composition. SRB-liposomes made of EggPC (curve 1) or DPhPC (curve 2) were resuspended in PBS and exposed to red light in the presence of 350 nM of **5**. Note SRB leakage from EggPC- but not from DPhPC-containing liposomes. Shown is one representative experiment out of three independent replicates.

Next, we investigated the role of lipid composition in liposome photodamage. The major component of egg yolk phosphatidylcholine (EggPC) is the lipid with single palmitate and single oleate residues, the latter moiety possessing one double bond. To compare the photosensitivity of unsaturated versus saturated lipids, we constructed the liposomes using a saturated composite. Phytanoic acid in diphytanoyl phosphatidylcholine (DPhPC) is saturated; therefore, its oxidative destruction is expected to be marginal, if any. We used this lipid because it is liquid at room temperature due to the branched phytanoic acid [30]. As shown in Figure 8, photoactivation of **5** led to fast dye leakage from liposomes made of unsaturated EggPC. In contrast, no photodamage was observed in liposomes containing the saturated lipid DPhPC under the same conditions. These data are in agreement with the fact that double bonds in fatty acid residues are most susceptible to oxidative lipid destruction [31].

**Figure 9:**
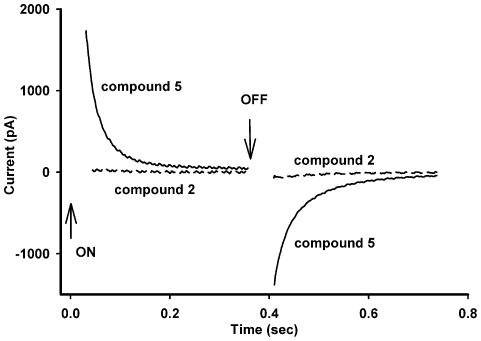
Differential permeability of a lipid membrane for compounds 2 and 5. Shown are time courses of electrical current after application of a voltage jump of *V* = 100 mV (at *t* = 0, ‘on’ response) in the presence of 10 nM of **5** or **2** and after switching off the voltage to zero (at *t* = 0.36 sec, ‘off’ response). Planar BLMs were formed from DPhPC (see MATERIALS AND METHODS). The experiments were performed in the buffer containing 10 mM Tris-HCl, pH 7.4 and 100 mM KCl. Shown is one representative experiment out of 3 with essentially the same results.

Finally, we evaluated the ability of **2** (cation) and **5** (anion) to permeate through artificial planar bilayer lipid membranes (BLM). Application of voltage to BLM doped with permeating ion led to appearance of electrical current that was maximal immediately after application of voltage (100 mV), and spontaneously decreased over time to zero (a current relaxation process in millisecond-to-second time scale [32], [33]. This relaxation is mechanistically linked to redistribution of anions/cations between two half-membrane leaflets after voltage application. Thus, permeating ions were depleted at one side of the membrane but accumulated at the other side. Figure 9 shows the current relaxation after a voltage jump of *V* = 100 mV and after switching off the voltage (‘off response’) for **5** (solid curve). In line with the model of redistribution of the anions across the membrane [32], the transient ‘off response’ resulted from backward translocation of **5** across the membrane interior, a rise of electrical current and an establishment of symmetry of concentrations of **5**. In contrast, compound **2** (Figure 9, dashed curve) or chlorin e6 (not shown) showed no measurable current relaxation responses at the same or higher concentrations. Thus, **5** permeated through BLM in the ionic form whereas **2** and chlorin e_6_ did not. These findings strongly suggest that a higher therapeutic potency of boronated compound **5** compared to boron-free **2** is associated with the ability of **5** to penetrate through lipid membranes.

## Discussion

In the present study we demonstrate that our novel boronated chlorin e_6_ derivative, chlorin e_6_ 13(1)-N-{2-[N-(1-carba-*closo*-dodecaboran-1-yl)methyl]aminoethyl}amide-15(2), 17(3)-dimethyl ester sodium salt (**5**) showed remarkable potency as a photosensitizer in two models of aggressive tumors. Studies of mechanisms of antitumor efficacy revealed that photoactivation of **5** triggered intracellular ROS generation followed by rapid (within the initial minutes after LI) damage of multiple membrane organelles, leading to cell necrosis. Most likely, this effect involved membrane permeabilization as demonstrated by the loss of integrity of artificial lipid vesicles upon photoactivation of **5**. Remarkably, the membrane photodamage and necrosis required nanomolar-to-submicromolar concentrations of novel photosensitizer. Also, the therapeutic effects in vivo were achievable at 5 mg/kg of **5**, a dose that caused no signs of general toxicity. Altogether, these results strongly suggest that **5** is a good candidate for further development as an antitumor photosensitizer.

Conjugation of boron cages to natural and synthetic tetrapyrrole containing compounds may increase the photodynamic potency [24]. In the present study the boronated derivative **5** demonstrated preferential efficacy in PDT of melanoma B16 and sarcoma M-1 compared with its boron-free counterpart **2**. One reason for this effect might be an elevated binding to albumin, a major carrier of many biomolecules [24]. However, the drug-albumin complex formation is not a conditio sine qua non for an elevated therapeutic potency of boronated chlorins because the binding constants of the complexes of albumin with **2** and **5** were similar (our unpublished observation). Furthermore, lipophilicity might be a prerequisite for design of therapeutically efficacious photosensitizers [34], [35]. Our measurements of lipophilicity showed that logP values for chlorin e_6_, **2** and **5** (determined as octanol-water partitioning) were very large (above 2.5). Thus, a higher in vivo antitumor efficacy of **5** is unlikely to be associated with lipophilicity.

Our photosensitizers targeted the cellular compartments enriched in hydrophobic environment. Interaction of the boronated derivative **5** with the cell was detectable immediately after the addition of the drug. Within 5 min, **5** entered the cells and accumulated in membrane organelles including the Golgi cysternae, ER, mitochondria and some lysosomes. According to these patterns of intracellular drug distribution, photoactivation of **5** followed by ROS generation caused damage to these organelles. A variety of the modes of death occur in PDT-treated cells, including apoptosis, autophagy and necrosis [17]. The apoptotic phenomena involving cytochrome c release, caspase activation and cleavage of critical cellular proteins can be detected several hours after photoactivation [36]–[38]. In contrast, we observed rapid (within the initial minutes after LI) loss of plasma membrane integrity in cells illuminated in the presence of **5** whereas nuclear fragmentation was absent. Although we cannot rule out other death-associated events associated with Δ*Ψ_m_* dissipation, the fact that permeability of the plasma membrane for PI occurred rapidly argues against apoptotic or autophagy pathways in our experimental systems. Thus, necrosis might be the leading mechanism of cell death upon photoactivation of novel boronated derivative of chlorin e_6_. The observations on cell lines corroborated the results of routine histological examination of PDT-treated B16 melanoma and M-1 sarcoma in which massive necrotic zones are detectable as early as 24 h post PDT (not shown). Other mechanisms of cell death may operate depending on the concentrations of **5**; however, photonecrosis was observed with nanomolar concentrations of this compound. We tend to consider necrosis an important mode of eradication of ‘intractable’ cancers [39] in which apoptotic pathways may be impaired in the course of tumor progression and/or as a result of preceding treatment. In these situations PDT would emerge as a therapeutic modality of choice.

Intracellular ROS generation is a prerequisite for photodynamic cell killing. In the present study ROS production in cells incubated with the boronated derivative **5** was detected by LI-induced burst of DCF fluorescence (i.e., oxidation of the probe DCFH); this burst was abrogated by sodium azide and trolox. Using silicon phthalocyanine Pc4 as a photosensitizer, a prolonged intracellular DCFH oxidation after PDT has been observed [40]. This effect might be attributed to long-lived lipid hydroperoxides most probably generated as secondary reactive species resulting from lipid oxidation by singlet oxygen. A 20–120 sec long development of the DCF fluorescence signal in Figures 3 and 5 can be also associated with the phenomenon of ROS-induced ROS release discovered by Zorov and colleagues [41]–[43].

The loss of the plasma membrane integrity, a hallmark of necrosis, can be mediated via energy starvation and subsequent disappearance of the ionic transmembrane gradient due to inactivation of ATP-dependent transporters [44], [45]. Alternatively, or in addition to, the plasma membrane might become permeable for PI-like compounds due to direct photodamage to membranes. Our data on liposomes loaded with the fluorescent dye demonstrated that LI in the presence of **5** led to rapid permeabilization of liposomal membranes. This observation is in line with the findings that chlorins can sensitize photoperoxidation of lipids and liposomal permeabilization [35], [46]. Recent results of Ehrenberg and colleagues highlighted the key role of lipid double bonds in photosensitized depolarization of liposomes [47]. Accordingly, the EggPC liposomes carrying fatty acids with double bonds were permeabilized upon photoactivation of **5** whereas the liposomes made of the saturated lipid DPhPC remained refractory. These results strongly suggest that plasma membrane permeabilization mediated by **5** is a result of sensitized lipid photooxidation.

Though **2** and **5** showed similar efficacy in provoking liposome permeabilization, the in vivo antitumor potency of **5** was much higher than that of **2**. This difference can be ascribed to the capability of **5** to permeate across a lipid membrane, the finding revealed in the present work. Taking into account that cellular membranes are asymmetric both in lipid and protein composition [48], one might suggest that the translocation of **5** from cis to trans membrane leaflet allows the drug to reach the targets in the plasma and/or intracellular membranes that are most susceptible to oxidative damage, e.g., if the trans leaflet is enriched in unsaturated lipids. This hypothesis is in agreement with the data of Ehrenberg and colleagues that the photodynamic potency of a photosensitizer essentially depends on the depth of its position in the membrane [20], [49], [50].

In summary, our novel boronated derivative of chlorin e_6_ demonstrated remarkable potency as a photosensitizer in cellular PDT and in animal models. At submicromolar concentrations this compound triggered intracellular ROS photogeneration followed by rapid injury of various cellular membranes. These events in combination with photodamage to artificial vesicles that contain an unsaturated lipid constituted necrosis as the leading mechanism of cell death upon light activation of the novel photosensitizer. The therapeutic effects, that is, regression of transplanted melanoma or sarcoma as well as the cure of animals, were achieved with no signs of general toxicity. Together, these results further support the proof of principle that boronation is a perspective direction for chemical modifications of chlorins aiming at more potent, well-tolerable antitumor photosensitizers.

## Materials and Methods

### Ethics

The verbal informed consent was obtained from all participants involved in this study. The written consent was not necessary since all participants worked as a multidisciplinary group and openly shared their results on the regular basis, from the study planning to the finalized manuscript. The research involving laboratory mice and rats has been approved by Institutional Review Board at Medical Radiological Research Center, Obninsk where the animals were hosted and all animal experiments performed. No specific approval was required for this particular study since in this institution the experiments with small laboratory animals were performed under the guidance of Ministry of Health and Social Development of Russian Federation, document No755 issued on 12.08.1977, and Declaration of Helsinki of World Medical Association (2000).

### Synthesis of photosensitizers

Chemical synthesis and characteristics of compounds are given in sections RESULTS and SUPPORTING INFORMATION.

### Animals and in vivo PDT

Six-to-eight week old (CBAxC57 BL6)F1 mice and 180–200 g weight rats were used in the experiments. For transplantation of murine B16 melanoma or rat M-1 sarcoma, tumor cells were freshly isolated from the tumor-bearing animal. One million B16 cells in 0.1 ml of PBS or 3×10^6^ sarcoma cells in 0.3 ml of PBS were injected under the skin of rear extremities (one inoculum per animal). After the tumor nodules reached ∼3–5 mm in diameter, animals were divided into 6 cohorts per each species (10 animals per group). Mice in the group 1 were injected with phosphate buffered saline pH 7.2 (PBS), mice in the group 2 were given LI in the absence of photosensitizers. Mice in groups 3–4 were injected with 5 mg/kg of either **2** or **5**, respectively; no LI was administered in these groups. Mice in groups 5–6 were injected with 5 mg/kg of **2** or **5**, respectively, followed by LI. Similar cohorts 1–6 were formed for rats bearing M-1 sarcoma transplants. PBS or photosensitizers were administered i.p. In preliminary experiments we found that the maximal accumulation of **5** in the tumors was detectable ∼1.5 hrs after i.p. injection. Therefore, we used a 1.5 h drug-light interval (i.e., the time between drug injection and tumor illumination). Hair around the tumor was epilated prior to LI. PDT was performed with the laser beam source Atcus-2 (Semi-Conductor Devices, St. Petersburg, Russian Federation; λ = 661 nm, density of light emission energy 150 J/cm^2^ for mice or 300 J/cm^2^ for rats). The duration of LI was calculated by the formula:
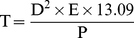
(1)where T is time (min.) of LI, D is the biggest diameter (cm) of the tumor, E is the density of absorbed light energy (J/cm^2^), and P is the power (mW) of emitted light.

The tumor size was measured immediately before LI and at days 3, 7, 14 and 21 post LI. The volume V (cm^3^) of the tumor was calculated using the equation:
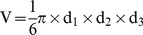
(2)where d_1,_ d_2_ and d_3_ are perpendicular diameters (cm) of the tumor mass.

Tumor bearing animals in the groups 1–4 were sacrificed by day 14 after PDT. Animals in the groups 5–6 were monitored for 30 days (mice) or 60 days (rats) post PDT. Tumor bearing animals in the groups 1–4 were sacrificed by day 14 after PDT. Animals in the groups 5–6 were monitored for 30 days (mice) or 60 days (rats) post PDT.

### Cell culture and dark cytotoxicity assays

The HCT116 human colon carcinoma McA 7777 rat liver epithelium, Rat-1 and REF fibroblasts, C6 rat glioma, MCF-10A human breast epithelium, HCT116 human colon carcinoma and MCF-7 human breast carcinoma cell lines (American Type Culture Collection, Manassas, VA) were propagated in Dulbecco's modified Eagle's medium supplemented with 5% fetal calf serum (Invitrogen, Carlsbad, CA), 2 mM *L*-glutamine, 100 units/ml penicillin, and 100 µg/ml streptomycin at 37°C, 5% CO_2_ in humidified atmosphere. Human lymphocytes were freshly isolated from peripheral blood of healthy donors. Newly synthesized compounds were dissolved to obtain 10 mM stock solutions in dimethyl sulfoxide, and serial aqueous dilutions were made immediately before experiments. The compounds were kept away from light. The experiments were performed in the dark. All chemicals were purchased from Sigma-Aldrich, St. Louis, MO unless specified otherwise. Dark cytotoxicity was tested in a 96-well format by reduction of 3-(4,5 dimethylthiazol-2-yl)-2,5-diphenyltetrazolium bromide to formazan (MTT-test) [51].

### PDT in cell culture and flow cytometry-assisted analysis of cell death

The C6, HCT116 or MCF-7 cells were plated overnight on a 35 mm Petri dish (Costar; 5×10^4^ cells per dish). Compound **5** (final concentration 0.1 µM) was added to the cells for 30 min. at 37°C, 5% CO_2_, then the medium was replaced with 0.5 ml PBS, and cell monolayers were illuminated for 10 min. using a monochromatic light source manufactured in Lebedev Institute of Physics, Moscow (λ = 633 nm, density of light emission energy 200 J/cm^2^). An aqueous solution of NaNO_2_ was placed between the light source and the cells to protect the monolayer from warming. After LI cells were replenished with fresh medium and kept at 37°C, 5% CO_2_. Control cells were left untreated (no drug, no light) or treated with 0.1 µM of **5** alone or illuminated in the absence of **5**. For quantitation of cells with damaged plasma membrane, PI (10 µg/ml) was added, and cells were analysed by flow cytometry on FL2 (FACSCalibur System, Becton Dickinson, USA).

### Fluorescence microscopy

Cells were grown on round 24 mm glass cover slips for 48 h to reach ∼50% confluency by the day of experiments. The concentrations of photosensitizers and duration of cell exposure are indicated in Results. To visualize the organelles, living cells were stained with the following fluorescent dyes (Invitrogen Corp., Carlsbad, CA): brefeldin A, BODIPY FL conjugate to visualize ER (0.2 µM, 5 min), BODIPY FL C_5_-ceramide, a marker of Golgi apparatus (5 µM, 15 min); MitoTracker Red CM-H_2_XRos, a dye sensitive to mitochondrial transmembrane electric potential *ΔΨm* (0.5 µM, 5 min) and lysosomal marker LysoTracker Green DND-26 (1 µM, 5 min). For detection of ROS, cells were loaded with 4 µM DCFH-DA for 5 min. PI (10 µg/ml) was used to detect necrotic cells. Digital images were acquired using an Axiovert 200M LSM-510 META microscope (Carl Zeiss AG, Germany). The 1024×1024 or 512×512 pixel confocal images were recorded with a Plan-Apochromat 63x/1.4 Oil DIC or Plan-Apochromat 100x/1.4 Oil Ph3 objective. Fluorescence of photosensitizers was excited with a 633 nm He–Ne laser, and emission was registered with the 650–710 nm band pass filter. For the 633 nm laser line the densities of light emission energy were 3.8 J/cm^2^ for 512×512 frame and 15.8 J/cm^2^ for 1024×1024 frame. Fluorescence of MitoTracker Red CM-H_2_XRos and PI was excited with a 543 nm He–Ne laser, and emission was registered with the 565–615 nm band pass filter. The 488 nm line of argon laser and 500–530 nm band pass filter were used to visualize the fluorescence of BODIPY FL C_5_-ceramide, brefeldin A, BODIPY FL conjugate, LysoTracker Green DND-26 and DCF. For simultaneous detection of the photosensitizer and organelles, two- or three-channel acquisition of a series of optical sections was done using a multi-track mode. The appropriate set of dichroic mirrors and band pass filters on the emission side were used. The pinholes for high-resolution images were set up according to the manufacturer's instructions. Zeiss LSM 510Meta Software release 3.2 was used for image processing and quantification.

In the experiments with sodium azide and trolox, the DCF fluorescence was examined by Zeiss Axiovert 200M epifluorescence microscope equipped with Plan-Apochromat 100x/1.4 Oil Ph3 objective and 450–490 nm excitation/515–565 nm emission filter set. Images were taken using an AxioCam Hrm digital camera (Carl Zeiss, Germany).

### Preparation of liposomes and SRB leakage assays

EggPC and DPhPC were purchased from Avanti Polar Lipids, Alabaster, AL. To prepare the liposomes, each lipid (5 mg) was dissolved in chloroform. After evaporation of the solvent, 0.5 ml of PBS containing 50 mM SRB was added, the suspension was vortexed for 2 min., then freeze-thawed 5 times with vortexing after each cycle and filtered through a 0.1 µM polycarbonate filter using a mini-extruder (Avanti Polar Lipids). SRB is more stable to photobleaching compared to carboxyfluorescein, the dye used in leakage assays. Liposomes were separated from the external dye by gel filtration on a Sephadex G-50 column, resuspended in PBS and illuminated with a xenon lamp (0.4 W/cm^2^) using a glass filter (λ>580 nm) to reduce SRB photobleaching. The photosensitizers and NaN_3_ were added to the suspension of liposomes immediately prior to illumination. Final concentrations of these agents are indicated in Results. As a control for the light-independent leakage of total dye content, liposomes were treated with 0.1% Triton X-100. Photodamage of liposomes was recorded by the increase of dye fluorescence in the external milieu after illumination. SRB fluorescence was measured on a Panorama Fluorat-02 spectrofluorimeter (Lumex, Moscow) at λ_excitation_ = 560 nm and λ_registration_ = 590 nm and expressed as arbitrary units or as the percentage to Triton X-100-induced dye leakage. In all experiments the lipid concentration was 5 µg/ml.

### Preparation of BLMs and electric current relaxation assays

BLMs were formed from a 2% solution of DPhPC (Avanti Polar Lipids, Alabaster, AL) in *n*-decane by the brush technique on a hole in a Teflon partition (0.8 mm in diameter) separating two compartments of a cell filled with buffer solutions [52]. Photosensitizers were added from stock solutions in DMSO to the bathing solutions at both sides of the BLM and incubated at least 10 min with constant stirring. The solution contained 10 mM Tris-HCl, pH 7.0; 10 mM 2-(*N*-morpholino)ethanesulfonic acid and 100 mM KCl. The electric currents (I) were recorded under voltage-clamp conditions. Voltages were applied to BLMs with Ag-AgCl electrodes placed directly into the cell. The currents measured with a patch-clamp amplifier (OES-2, OPUS, Moscow) were digitized (NI-DAQmx, National Instruments, Austin, TX) and analyzed using WinWCP Strathclyde Electrophysiology Software (designed by J. Dempster, University of Strathclyde, UK). In the current relaxation experiments the voltage was switched from zero to 100 mV at *t* = 0 and the current across the membrane (I(*t*)) started to decrease from the initial level I(0) to steady-state level I(∞). The capacitance response of the unmodified membrane (current after voltage jump, control) was recorded at the beginning of each experiment. The records in the presence of chlorin e_6_, **2** or **5** was analyzed after subtraction of control records.

### Statistical analysis

Student's *t*-test was used for statistical analysis of data.

## Supporting Information

File S1 1H and 11B NMR spectra were recorded on a Bruker Avance-400 spectrometer in (CD3)2CO. Chemical shifts (δ) are given in ppm relative to internal chloroform. IR spectra were recorded on a Specord M-82 spectrometer (Carl Zeiss) in KBr tablets. The UV-Vis spectra were measured on a Jasco UV 7800 spectrophotometer in CHCl3. Mass spectra were obtained using Vision 2000 (MALDI) mass spectrometer, the most intense peaks are given below for each compound. Merck silica gel L 0.040–0.08 mesh was used for column chromatography. The identities of new compounds were verified on TLC 60 F254 plates (Merck) in CHCl3-MeOH (9∶1 v/v) solvent system. The solvents were purified according to standard procedures. Cesium salt of chlorin e6 13(1)-N-{2-[N-(1-carba-closo-dodecaboran-1-yl) methyl] aminoethyl}amide-15(2), 17(3)-dimethyl ester (4). A solution of 2 (200 mg, 0.3 mmol) and 1-trifluoromethanesulfonylmethyl-1-carba-closo-dodecaborate cesium 3 (184 mg, 0.43 mmol) in 10 ml of THF was stirred with 2 ml of BSA at room temperature for 1 h. The reaction mixture was diluted with CHCl3 (50 ml), washed with 3% aqueous CsCl solution (3×30 ml), dried over Na2SO4 and evaporated to dryness in vacuo. The crude product was purified by column chromatography on SiO2 using CHCl3-MeOH (9∶1) as eluent to give 203 mg (71%) of pure compound 4. IR (KBr), n (cm-1): 2530 (BH), 1722 (C = O, ester), 1609 (chlorin band), 1656 (amide I), 1520 (amide II). UV-Vis (CHCl3, lmax, nm, e.10-3), 404 (51.4), 501 (9.40), 610 (2.6), 663 (35.9). 1H NMR (acetone-d6), d, ppm: 9.79 (s, 1H, 10-H); 9.76 (s, 1H, 5-H); 9.11 (s, 1H, 20-H); 8.24 (dd, 1H, J = 17.3 and 11.5 Hz, 3(1)-H); 6.42 (d, 1H, J = 18.3 Hz, 3(2)-H(trans)); 6.16 (d, 1H, J = 12.4 Hz, 3(2)-H (cis)); 15(1)-CH2: 5.53 (d, 1H, J = 18.5 Hz) and 5.36 (d, 1H, J = 18.7 Hz); 4.67 (q, 1H, J = 7.5 Hz, 18-H); 4.51 (br. d., 1H, J = 8.6 Hz, 17-H); 4.13 (br. s., 1H, 13(1)-NH); 3.80 (q, 2H, J = 7.5 Hz, 8(1)-CH2); 3.75 (m, 4H, 13(2)-CH2, 13(3)-CH2); 3.70 (s, 3H, 15(3)-CH3); 3.61 (s, 3H, 17(4)-CH3); 3.60 (s, 3H, 12(1)-CH3); 3.53 (s, 3H, 2(1)-CH3); 3.32 (s, 3H, 7(1)-CH3); 2.82 (s, 1H, 13(3)-NH); 2.52 (s, 2H, CH2-carborane); 2.28 (m, 4H, 17(1)-CH2 and 17(2)-CH2)); 3.0-1.4 (m., 11H, BH), 1.71 (d, 3H, J = 7.1 Hz, 18(1)-CH3), 0.88 (t, 3H, J = 7.5 Hz, 8(2)-CH3); -1.53 (br. s., 1H, NH); -1.82 (br. s., 1H, NH). 11B NMR (acetone-d6), d, ppm.: -9.02 (d, 1B, J = 136 Hz, B(12)); -13.15 (d, 5B, J = 130 Hz, B(2-6)); -14.37 (d, 5B, J = 154 Hz, B(7-11)). Mass spectra, (m/z): 821 [M - Cs+]-. Sodium salt of chlorin e6 13(1)-N-{2-[N-(1-carba-closo-dodecaboran-1-yl) methyl] aminoethyl}amide-15(2), 17(3)-dimethyl ester (5). A solution of compound 4 (40 mg, 0.04 mmol) in 100 ml MeCN was passed through column with ion-exchange resin DOWEX 50 WX8-200 (2×5 cm). The solvent was evaporated to 50 ml in vacuo, and the concentrated solution was again passed through the column with ion-exchange resin until the completion of cation exchange registered by TLC. After the evaporation of solvent in vacuo, 33.5 mg (99.5%) of compound 5 was obtained. IR spectra (KBr), n (cm-1): 2528 (BH), 1721 (C = O, ester), 1606 (chlorin band), 1657 (amide I), 1519 (amide II). UV-Vis spectra (CHCl3, lmax, nm, e.10-3): 406 (58.0), 503 (10.6), 610 (2.9), 663 (40.5). 1H NMR (acetone-d6), d, ppm.: 9.78 (s, 1H, 10-H); 9.75 (s, 1H, 5-H); 9.10 (s, 1H, 20-H); 8.23 (dd, 1H, J = 17.3 and 11.5 Hz, 3(1)-H); 6.43 (d, 1H, J = 18.3 Hz, 3(2)-H(trans)); 6.16 (d, 1H, J = 12.4 Hz, 3(2)-H (cis)); 15(1)-CH2: 5.51 (d, 1H, J = 18.4 Hz) and 5.34 (d, 1H, J = 18.6 Hz); 4.67 (q, 1H, J = 7.5 Hz, 18-H); 4.51 (br. d., 1H, J = 8.6 Hz, 17-H); 4.13 (br. s., 1H, 13(1)-NH); 3.81 (q, 2H, J = 7.5 Hz, 8(1)-CH2); 3.75 (m, 4H, 13(2)-CH2, 13(3)-CH2); 3.74 (s, 3H, 15(3)-CH3); 3.61 (s, 3H, 17(4)-CH3); 3.60 (s, 3H, 12(1)-CH3); 3.52 (s, 3H, 2(1)-CH3); 3.32 (s, 3H, 7(1)-CH3); 2.82 (s, 1H, 13(3)-NH); 2.52 (s, 2H, CH2-carborane); 2.28 (m, 4H, 17(1)-CH2 and 17(2)-CH2)); 3.0-1.4 (m., 11H, BH), 1.71 (d, 3H, J = 7.1 Hz, 18(1)-CH3), 0.87 (t, 3H, J = 7.4 Hz, 8(2)-CH3); -1.55 (br. s., 1H, NH); -1.84 (br. s., 1H, NH). 11B NMR (acetone-d6), d, ppm.: -9.00 (d, 1B, J = 136 Hz, B(12)); -13.13 (d, 5B, J = 131 Hz, B(2-6)); -14.35 (d, 5B, J = 155 Hz, B(7-11)). Anal. calcd. for C40H58B11N6NaO5: C, 56.87; H, 6.87; B, 14.08. Found: C, 56.77; H, 6.92; B, 14.01. Mass spectra, (m/z): 821 [M - Na+]-.
(0.03 MB DOC)

Figure S1 Synthesis of photosensitizers.
(0.51 MB TIF)

Figure S2 Measurements of quantum yields of singlet oxygen. Shown are time courses of DMA fluorescence at 426 nm upon continuous light exposure (680 nm laser diode) in the presence of 1 µM of ZnPcS4, 2, 5 or chlorin e6. See text for details.
(2.09 MB TIF)
