# Supplementary material for: Novel Photosensitizers Trigger Rapid Death of Malignant Human Cells and Rodent Tumor Transplants via Lipid Photodamage and Membrane Permeabilization
Source: PLoS One. 2010 Sep 15;5(9):e12717. doi: 10.1371/journal.pone.0012717 (PMC2939899; doi:10.1371/journal.pone.0012717)
Supplement: File S1 — 1H and 11B NMR spectra were recorded on a Bruker Avance-400 spectrometer in (CD3)2CO. Chemical shifts (δ) are given in ppm relative to internal chloroform. IR spectra were recorded on a Specord M-82 spectrometer (Carl Zeiss) in KBr tablets. The UV-Vis spectra were measured on a Jasco UV 7800 spectrophotometer in CHCl3. Mass spectra were obtained using Vision 2000 (MALDI) mass spectrometer, the most intense peaks are given below for each compound. Merck silica gel L 0.040–0.08 mesh was used for column chromatography. The identities of new compounds were verified on TLC 60 F254 plates (Merck) in CHCl3-MeOH (9∶1 v/v) solvent system. The solvents were purified according to standard procedures. Cesium salt of chlorin e6 13(1)-N-{2-[N-(1-carba-closo-dodecaboran-1-yl) methyl] aminoethyl}amide-15(2), 17(3)-dimethyl ester (4). A solution of 2 (200 mg, 0.3 mmol) and 1-trifluoromethanesulfonylmethyl-1-carba-closo-dodecaborate cesium 3 (184 mg, 0.43 mmol) in 10 ml of THF was stirred with 2 ml of BSA at room temperature for 1 h. The reaction mixture was diluted with CHCl3 (50 ml), washed with 3% aqueous CsCl solution (3×30 ml), dried over Na2SO4 and evaporated to dryness in vacuo. The crude product was purified by column chromatography on SiO2 using CHCl3-MeOH (9∶1) as eluent to give 203 mg (71%) of pure compound 4. IR (KBr), n (cm-1): 2530 (BH), 1722 (C = O, ester), 1609 (chlorin band), 1656 (amide I), 1520 (amide II). UV-Vis (CHCl3, lmax, nm, e.10-3), 404 (51.4), 501 (9.40), 610 (2.6), 663 (35.9). 1H NMR (acetone-d6), d, ppm: 9.79 (s, 1H, 10-H); 9.76 (s, 1H, 5-H); 9.11 (s, 1H, 20-H); 8.24 (dd, 1H, J = 17.3 and 11.5 Hz, 3(1)-H); 6.42 (d, 1H, J = 18.3 Hz, 3(2)-H(trans)); 6.16 (d, 1H, J = 12.4 Hz, 3(2)-H (cis)); 15(1)-CH2: 5.53 (d, 1H, J = 18.5 Hz) and 5.36 (d, 1H, J = 18.7 Hz); 4.67 (q, 1H, J = 7.5 Hz, 18-H); 4.51 (br. d., 1H, J = 8.6 Hz, 17-H); 4.13 (br. s., 1H, 13(1)-NH); 3.80 (q, 2H, J = 7.5 Hz, 8(1)-CH2); 3.75 (m, 4H, 13(2)-CH2, 13(3)-CH2); 3.70 (s, 3H, 15(3)-CH3); 3.61 (s, [file pone.0012717.s001.doc]

# SUPPORTING INFORMATION

^1^H and ^11^B NMR spectra were recorded on a Bruker Avance-400 spectrometer in (CD_3_)_2_CO. Chemical shifts (δ) are given in ppm relative to internal chloroform. IR spectra were recorded on a Specord M-82 spectrometer (Carl Zeiss) in KBr tablets. The UV–Vis spectra were measured on a Jasco UV 7800 spectrophotometer in CHCl_3_. Mass spectra were obtained using Vision 2000 (MALDI) mass spectrometer, the most intense peaks are given below for each compound. Merck silica gel L 0.040–0.08 mesh was used for column chromatography. The identities of new compounds were verified on TLC 60 F254 plates (Merck) in CHCl_3_–MeOH (9:1 v/v) solvent system. The solvents were purified according to standard procedures.

**Cesium salt of chlorin e_6_ 13(1)-N-{2-[N-(1-carba-*closo*-dodecaboran-1-yl) methyl] aminoethyl}amide-15(2), 17(3)-dimethyl ester (4)**. A solution of **2** (200 mg, 0.3 mmol) and 1-trifluoromethanesulfonylmethyl-1-carba-*closo*-dodecaborate cesium **3** (184 mg, 0.43 mmol) in 10 ml of THF was stirred with 2 ml of BSA at room temperature for 1 h. The reaction mixture was diluted with CHCl_3_ (50 ml), washed with 3% aqueous CsCl solution (3 x 30 ml), dried over Na_2_SO_4_ and evaporated to dryness in vacuo. The crude product was purified by column chromatography on SiO_2_ using CHCl_3_–MeOH (9:1) as eluent to give 203 mg (71%) of pure compound **4**. IR (KBr), ν (см^-1^): 2530 (ВН), 1722 (C=O, ester), 1609 (chlorin band), 1656 (amide I), 1520 (amide II). UV-Vis (CHCl_3_, λ_max_, nm, ε.10^-3^), 404 (51.4), 501 (9.40), 610 (2.6), 663 (35.9). ^1^Н NMR (acetone-d_6_), δ, ppm: 9.79 (s, 1Н, 10-Н); 9.76 (s, 1Н, 5-Н); 9.11 (s, 1Н, 20-Н); 8.24 (dd, 1Н, J = 17.3 and 11.5 Hz, 3(1)-Н); 6.42 (d, 1Н, J = 18.3 Hz, 3(2)-H(trans)); 6.16 (d, 1Н, J = 12.4 Hz, 3(2)-Н (cis)); 15(1)-СН_2_: 5.53 (d, 1Н, J = 18.5 Hz) and 5.36 (d, 1Н, J = 18.7 Hz); 4.67 (q, 1Н, J = 7.5 Hz, 18-Н); 4.51 (br. d., 1Н, J = 8.6 Hz, 17-Н); 4.13 (br. s., 1Н, 13(1)-NH); 3.80 (q, 2Н, J = 7.5 Hz, 8(1)-СН_2_); 3.75 (m, 4Н, 13(2)-СН_2_, 13(3)-СН_2_); 3.70 (s, 3Н, 15(3)-СН_3_); 3.61 (s, 3Н, 17(4)-СН_3_); 3.60 (s, 3Н, 12(1)-СН_3_); 3.53 (s, 3Н, 2(1)-СН_3_); 3.32 (s, 3Н, 7(1)-СН_3_); 2.82 (s, 1H, 13(3)-NH); 2.52 (s, 2H, СН_2_-carborane); 2.28 (m, 4Н, 17(1)-СН_2_ and 17(2)-СН_2_)); 3.0-1.4 (m., 11Н, ВН), 1.71 (d, 3Н, J = 7.1 Hz, 18(1)-СН_3_), 0.88 (t, 3Н, J = 7.5 Hz, 8(2)-CH_3_); -1.53 (br. s., 1Н, NH); -1.82 (br. s., 1Н, NH). ^11^В NMR (acetone-d_6_), δ, ppm.: -9.02 (d, 1В, J = 136 Hz, В(12)); -13.15 (d, 5В, J=130 Hz, В(2-6)); -14.37 (d, 5В, J=154 Hz, В(7-11)). Mass spectra, (*m/z*): 821 [М - Сs^+^]^-^.

# Sodium salt of chlorin e_6_ 13(1)-N-{2-[N-(1-carba-*closo*-dodecaboran-1-yl) methyl] aminoethyl}amide-15(2), 17(3)-dimethyl ester (5). A solution of compound 4 (40 mg, 0.04 mmol) in 100 ml MeCN was passed through column with [**ion-exchange resin**](http://www.multitran.ru/c/m.exe?t=355262_1_2) DOWEX 50 WX8-200 (2х5 cm). The solvent was evaporated to 50 ml in vacuo, and the concentrated solution was again passed through the column with [**ion-exchange resin**](http://www.multitran.ru/c/m.exe?t=355262_1_2) until the completion of cation exchange registered by TLC. After the evaporation of solvent in vacuo, 33.5 mg (99.5%) of compound 5 was obtained. IR spectra (KBr), ν (см^-1^): 2528 (ВН), 1721 (C=O, ester), 1606 (chlorin band), 1657 (amide I), 1519 (amide II). UV-Vis spectra (CHCl_3_, λ_max_, nm, ε.10^-3^): 406 (58.0), 503 (10.6), 610 (2.9), 663 (40.5). ^1^Н NMR (acetone-d_6_), δ, ppm.: 9.78 (s, 1Н, 10-Н); 9.75 (s, 1Н, 5-Н); 9.10 (s, 1Н, 20-Н); 8.23 (dd, 1Н, J = 17.3 and 11.5 Hz, 3(1)-Н); 6.43 (d, 1Н, J = 18.3 Hz, 3(2)-H(trans)); 6.16 (d, 1Н, J = 12.4 Hz, 3(2)-Н (cis)); 15(1)-СН_2_: 5.51 (d, 1Н, J = 18.4 Hz) and 5.34 (d, 1Н, J = 18.6 Hz); 4.67 (q, 1Н, J = 7.5 Hz, 18-Н); 4.51 (br. d., 1Н, J = 8.6 Hz, 17-Н); 4.13 (br. s., 1Н, 13(1)-NH); 3.81 (q, 2Н, J = 7.5 Hz, 8(1)-СН_2_); 3.75 (m, 4Н, 13(2)-СН_2_, 13(3)-СН_2_); 3.74 (s, 3Н, 15(3)-СН_3_); 3.61 (s, 3Н, 17(4)-СН_3_); 3.60 (s, 3Н, 12(1)-СН_3_); 3.52 (s, 3Н, 2(1)-СН_3_); 3.32 (s, 3Н, 7(1)-СН_3_); 2.82 (s, 1H, 13(3)-NH); 2.52 (s, 2H, СН_2_-carborane); 2.28 (m, 4Н, 17(1)-СН_2_ and 17(2)-СН_2_)); 3.0-1.4 (m., 11Н, ВН), 1.71 (d, 3Н, J = 7.1 Hz, 18(1)-СН_3_), 0.87 (t, 3Н, J = 7.4 Hz, 8(2)-CH_3_); -1.55 (br. s., 1Н, NH); -1.84 (br. s., 1Н, NH). ^11^В NMR (acetone-d_6_), δ, ppm.: -9.00 (d, 1В, J = 136 Hz, В(12)); -13.13 (d, 5В, J=131 Hz, В(2-6)); -14.35 (d, 5В, J=155 Hz, В(7-11)). Anal. calcd. for C_40_H_58_B_11_N_6_NaO_5_: C, 56.87; H, 6.87; B, 14.08. Found: C, 56.77; H, 6.92; B, 14.01. Mass spectra, (*m/z*): 821 [М - Na^+^]^-^.
