# Supplementary figures and images for: Novel Photosensitizers Trigger Rapid Death of Malignant Human Cells and Rodent Tumor Transplants via Lipid Photodamage and Membrane Permeabilization
Source: PLoS One. 2010 Sep 15;5(9):e12717. doi: 10.1371/journal.pone.0012717 (PMC2939899; doi:10.1371/journal.pone.0012717)

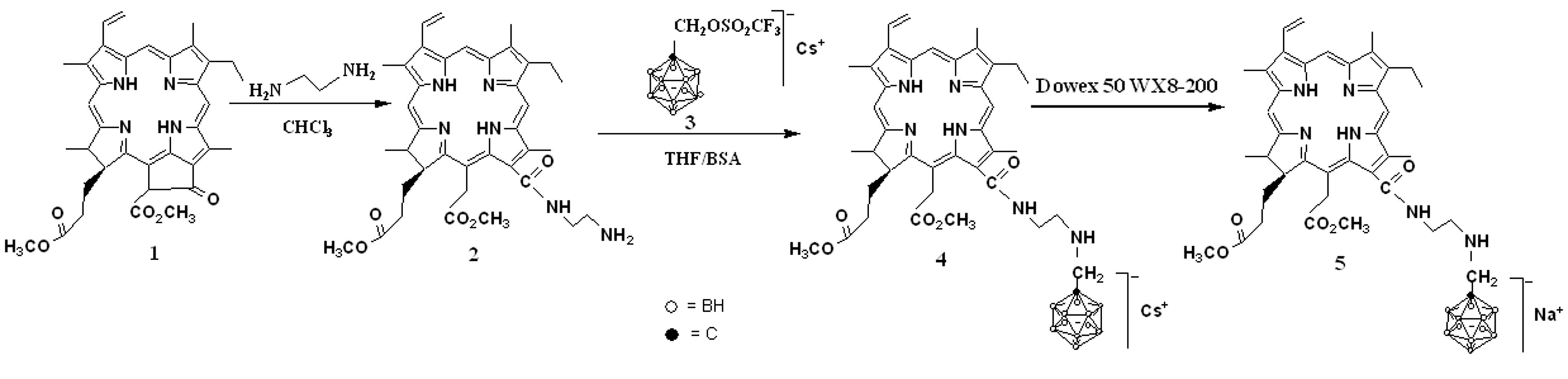

Supplement: Figure S1 — Synthesis of photosensitizers. (0.51 MB TIF) [file pone.0012717.s002.tif]

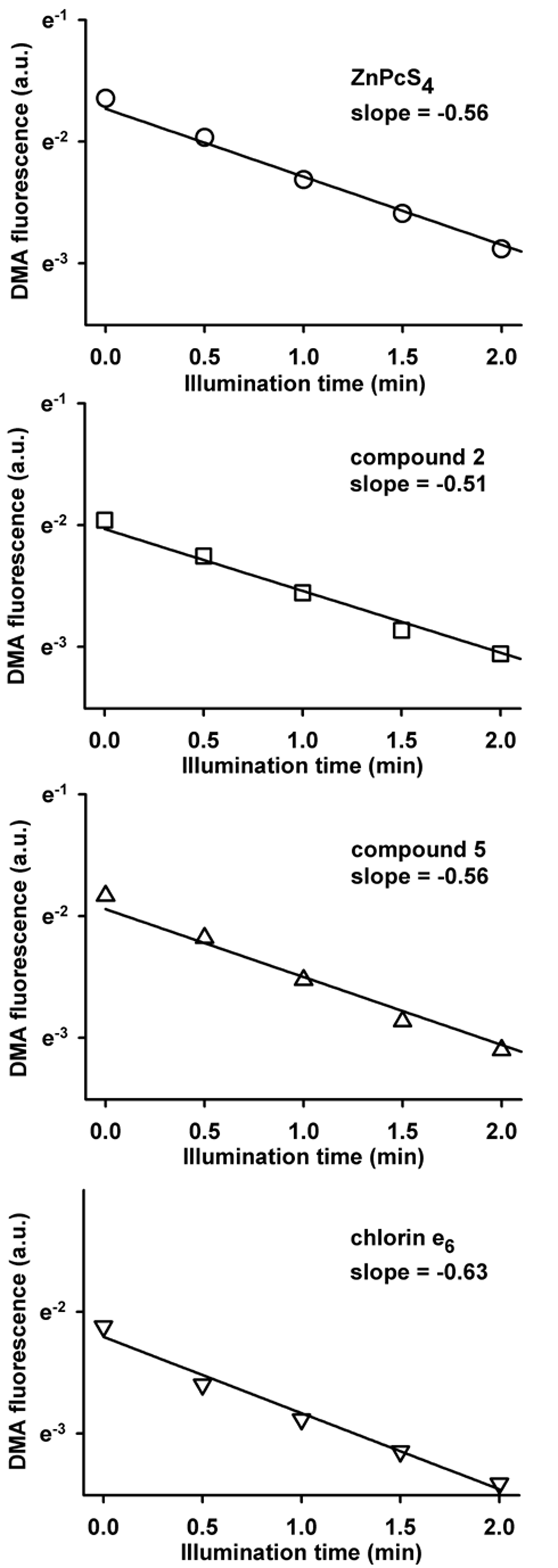

Supplement: Figure S2 — Measurements of quantum yields of singlet oxygen. Shown are time courses of DMA fluorescence at 426 nm upon continuous light exposure (680 nm laser diode) in the presence of 1 µM of ZnPcS4, 2, 5 or chlorin e6. See text for details. (2.09 MB TIF) [file pone.0012717.s003.tif]
